# CART Peptides Regulate Psychostimulants and May be Endogenous Antidepressants

**DOI:** 10.2174/157015911795017074

**Published:** 2011-03

**Authors:** Job M.O, McNamara I.M, Kuhar M.J

**Affiliations:** Yerkes National Primate Research Center of Emory University, 954 Gatewood Rd NE, Atlanta GA 30329, USA

**Keywords:** CART peptide, antidepressant, depression, forced swim test, anxiety, arousal, drug abuse.

## Abstract

CART peptides are endogenous neurotransmitters that are involved in a variety of physiologic functions. Injection of CART 55-102 into the nucleus accumbens produces no effect, but when co-administered with cocaine, it reduces the locomotor and rewarding properties of cocaine. In a human study, subjects carrying a missense mutation of the CART gene exhibited increased anxiety and depression. Also, several animal studies support the idea that CART is involved in anxiety and depression, and they also suggest several possible mechanisms by which this may occur. Thus, there is interesting evidence that CART peptides play a role in anxiety and depression, and that CART peptides may be endogenous antidepressants.

## INTRODUCTION

CART (cocaine and amphetamine regulated transcript) peptides are peptide neurotransmitters and neurohormones involved in the effects of psychostimulants as well as in other functions [1-3]. CART peptides appear to attenuate the actions of psychostimulants at several sites in the brain. For example, they blunt the locomotor actions of cocaine and amphetamine and also the rewarding and reinforcing properties of cocaine when injected into the nucleus accumbens [4-7]. 

In the last several years, new data suggests that CART peptides (CART 55-102 and 62-102) may have antidepressant properties, and some aspects of this are especially intriguing. An interesting case involves an Italian family that has been identified with a missense mutation in the CART gene which results in missorted and improperly processed CART peptide [8, 9]. The striking phenotype of family members carrying the mutation is early onset obesity. These findings have contributed substantially to our understanding of the role of CART peptides in feeding, and of the processing of these peptides. Moreover, the availability of this family offers a unique opportunity to explore other roles of CART peptides in humans. Accordingly, several family members underwent psychological testing.

**Fig. (1):**
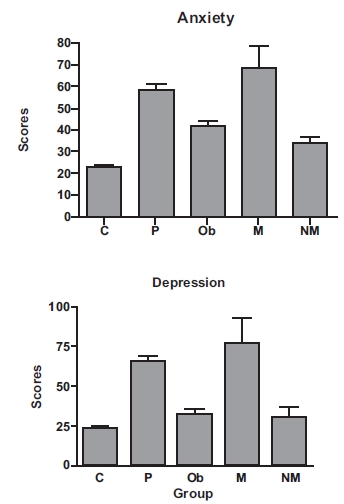
Anxiety and Depression scores among family members, both with and without the mutation, and other groups. C shows the scores for the control (normal) group (n = 188 for the depression control group, and n = 210 for the anxiety control group). P represents the pathological subjects (n = 20 for depression, and n = 35 for anxiety). Ob indicates obese children from the same clinic but without the mutation and from other families (n = 30 in both cases). M indicates scores for family members carrying the mutation (n = 3), and NM shows data from the family members without the mutation (n = 2). See text for details and results of ANOVA analysis. Reproduced from [11].

The test utilized was developed by Italian psychologists for Italian children and adolescents, and evaluates levels of depression, anxiety and eating disorders [10] (also see [11] for details). From the family of interest, only three family members with the mutation took the test, and two family members without the mutation were tested. The control populations were much larger. The family members carrying the mutation (M) scored high on the anxiety and depression scales, in comparison to members of the family without the mutation (NM), a large normal group (C), or a group of obese children from the same clinic but without the mutation (Ob) (Fig. **1**). Their anxiety and depression scores were similar to the scores of other anxious and depressed patients (P), and it is known that anxiety and depression can co-occur.

Among the anxiety scores (Fig. **1**), C was different from M, Ob and P (P<0.001). Also, P was different from Ob (P<0.001), and Ob was different from M (P<0.01). Importantly, M was different from NM (P<0.05). Thus, the M group, or the family members carrying the mutation, was different from all groups except the P (pathological) group. Among the depression scores, C was different from P and M (P<0.001) and from Ob (P<0.01) as well. P was different from Ob (P<0.001) and NM (P<0.01). Ob was also different from M (P<0.001), and importantly, M was different from NM (P<0.01). 

While the obese patients from the same clinic (Ob) had scores significantly higher compared to normals (C), the scores from the M group were even higher compared to the Ob group. This suggests that the obese phenotype by itself is not the sole determinant of the high scores. However, it must be kept in mind that these data reflect very small numbers of subjects for both the NM (n = 2) and N (n = 3) groups. The other family members did not participate, and there are no other known groups of humans with this or a similar mutation. Thus, a cautious interpretation is needed, and further studies of CART and depression are warranted. Also, in this human study, it is not known if the anxiety and depression effects are a direct or indirect result of the mutation.

## IS THE CART PEPTIDE AN ENDOGENOUS ANTIDEPRESSANT?

Given these findings in humans, it is interesting that several studies in animals have provided supportive results. CART peptides are found in brain regions and tissues involved in depression, anxiety and stress [3, 12-14]. There is data showing that CART mRNA is down regulated in the frontal cortex of rats subjected to chronic mild stress: an animal model of depression [15]. Several tests with rodents including elevated plus maze, social interaction, and startle suggest a role for CART peptides in anxiety and arousal [16-18]. A recent study by Dandekar and colleagues used social isolation and olfactory bulbectomy models of depression to explore the role of CART peptides in depression [19]. They found that the social isolation-induced and bulbectomy-induced depression-like behaviors were reversed following acute or subchronic treatment with CART peptide. Also, an important finding was that CART peptide, given intracerebroventricularly (icv) by itself in normal animals, reduced immobility time in the forced swim test, and therefore CART peptide itself seems to have antidepressant properties. Similar results were obtained when the target of injection was the central nucleus of the amygdala. 

Stress and depression are thought to be linked. The hypothalamic-pituitary-adrenal (HPA) axis play a role in anxiety and depression [20]. During stress, the HPA-axis is activated and corticotrophin releasing factor (CRF) is released to stimulate the release of adrenocorticotropin hormone (ACTH) from the anterior pituitary, which in turn stimulates the release of glucocorticoids from the adrenal glands. Stress regulates CART expression in parts of the hypothalamus [21,  22], perhaps through several mechanisms. CRF regulates CART peptide release in the HPA-axis [23], and vice versa [24]. Approximately 60% of CART varicosities in the hypothalamic paraventricular nucleus (PVN) were shown to thalamic paraventricular nucleus (PVN) were shown to be in juxtaposition to CRF-containing neurons [25], and CART peptides affect the HPA axis through CRF activation [24]. Moreover, glucocorticoids regulate CART levels in plasma [26] and in the brain [27], and CART has been suggested to be involved in the HPA-axis stress response [28]. 

The hypothalamic-pituitary-thyroid (HPT)-axis is also thought to play a role in the pathophysiology of depression [29]. Thyrotropin-releasing hormone-(TRH) has been shown to have antidepressant properties [30-32], and TRH-1 and -2 receptor deficient mice display an increased depression-like behavioral phenotype [33, 34]. Electroconvulsive therapy, which is an effective treatment of depression, increases TRH gene expression in the brain [35, 36], and the CART system regulates TRH activity [37-39]. This is supported by the observation that CART-containing neurons densely innervate, and are co-localized with, the majority of hypophysiotropic TRH neurons in the PVN [40-42]. Furthermore, CART activity increased the mRNA and peptide levels of TRH in TRH-containing hypophysiotropic neurons, suggesting that CART peptides exerts a stimulatory effect on TRH [38]. Also, Stanley *et al*. showed that CART peptides stimulate the release of TRH [39] from hypothalamic explants, and Brunetti *et al*. presented data showing that CART and TRH systems seem to exert a synergistic effect [43] (in inhibiting dopamine release from the hypothalamus). It is therefore conceivable that CART may be exerting antidepressant effects partly due to its involvement in the regulation of TRH.

About 37% of CART neurons in the magnocellular PVN co-localize with oxytocin [44], suggesting that CART may regulate oxytocin activity. Indeed, Vrang *et al*. showed that CART activity stimulates oxytocin release from the hypothalamus [45]. Oxytocin has antidepressant properties and has been proposed to be involved in the mechanisms of action of some antidepressants [46, 47]. Oxytocin regulates the HPA-axis and CART-mediated regulation of oxytocin activity indicates that CART may exert antidepressant properties partly through this mechanism. 

The hypothalamic-pituitary-somatotrophic (HPS) axis, like the HPA- and HPT- axes (discussed above) is also thought to be involved in the etiology of depression [48], and the CART system regulates the HPS axis. For example, CART peptide altered growth hormone (GH) release [39, 49] and interestingly, in the hypothalamic periventricular nucleus (PeV), approximately one-third of somatostatin cells were CART-immunoreactive [50, 51], suggesting that CART and somatostatin may regulate each other. Somatostatin has anti-depressant properties [52, 53]; antidepressants alter somatostatin pharmacology within the brain [54, 55], somatostatin mRNA is decreased in the hypothalamic PeV in a rats subjected to chronic mild stress (a model of depression) [56], and repeated electroconvulsive therapy increases somatostatin expression in the hippocampus [57]. The interaction between the CART system and the HPS-axis suggests that CART plays a role in depression. 

Thus, CART peptides are heavily involved in hypothalamic mechanisms and may exert an antidepressant action through these mechanisms. But CART can have antidepressant effects through other mechanisms and brain regions as well. Many antidepressants increase serotonin (5HT) which produces many effects some of which are antidepressant. It was shown that injection of CART 61-102 into the dorsal raphe nucleus increased 5HT efflux as determined by *in vivo* microdialysis suggesting that CART peptide may exert an antidepressant effect *via *serotonergic mechanisms [58]. 

Interestingly, a recent study showed that repeated electroconvulsive shock (an effective treatment for depression in humans), increased CART mRNA and CART protein in the rat nucleus accumbens [59], a brain region involved in reward. Further, it has been proposed that BDNF (brain-derived neurotrophic factor) is required for antidepressant-induced behavioral effects [60-62], and CART peptides have been shown to increase BDNF mRNA in hippocampal neurons [63]. Taken together, these data suggest that CART may behave like an antidepressant; however, more experiments are necessary to determine the precise role of CART in modulating depression-like behaviors.

There are some other interesting observations. CART 55-102 produces arousal and antidepressant-like behavior at doses lower than those required for other physiologic effects. For example, CART peptide levels in icv injections that produce antidepressant-like effects were in the 25-100 ng range, while doses for anti-feeding effects are in the 1-2 ug range [64]. Also, CART peptide can produce anxiogenic or arousal effects in doses that are less than 1 ug [17]. Thus, the most potent effects of CART peptides may have to do with anxiety and depression.

Another interesting observation is a connection with drug abuse in humans. Human drug abuse often shows a co-occurrence with anxiety and depression (see [65] for references). It is interesting that CART peptides represents molecules that affect both drug intake [4] and depression and anxiety, as summarized above. This is not to suggest that the co-occurrence of drug use and mood disorders is due solely to a problem with CART peptides, but the CART system is likely to be an important factor in drug abusing populations.

Data from humans and animals suggest that CART is associated with depression. As summarized above, there are several potential mechanisms by which CART may change behaviors and neural circuitry associated with depression . Thus, the CART system is a reasonable target for antidepressant and anti-anxiety drug discovery [14, 66]. A more precise determination of the role of CART in depression will require a better understanding of depression itself. Also, because of its role in drug abuse, CART-derived medications may be useful in a drug abuse setting.
